# Actual Conditions and Implementation-Related Factors of Activities to Prevent Disability in Multi-Area of Daily Function, Nutritional Status, and Oral Function among Community-Dwelling Older Adults

**DOI:** 10.31662/jmaj.2025-0558

**Published:** 2026-03-27

**Authors:** Keita Nakagawa, Hungu Jung, Hitoshi Okamura, Tomoyuki Ishii, Shinya Ishii

**Affiliations:** 1 Faculty of Health Sciences, Hiroshima Cosmopolitan University, Hiroshima, Japan; 2 Department of Medicine for Integrated Approach to Social Inclusion, Graduate School of Biomedical and Health Sciences, Hiroshima University, Hiroshima, Japan; 3 Department of Psychosocial Rehabilitation, Graduate School of Biomedical and Health Sciences, Hiroshima University, Hiroshima, Japan; 4 Maple Hill Hospital, Medical Corporation Tijinkai, Otake, Japan

**Keywords:** older adults, community residents, disability, frailty, prevention activities

## Abstract

**Introduction:**

Implementing multi-area disability prevention activities is an effective and efficient strategy for preventing the transition to frailty and the need for long-term care among older adults. In this study, we aimed to investigate the actual conditions and factors associated with implementing these prevention activities in daily function, nutritional status, and oral function.

**Methods:**

In this cross-sectional study, a mailed questionnaire was sent to 1,800 randomly selected older adults. Ten locations were selected to minimize bias in population characteristics, including urban, rural, and mountainous areas. The questionnaire included items on the actual conditions of disability prevention activities concerning daily function, nutritional status, and oral function; their content and frequency of implementation; and variables considered to be associated with the total number of activities. Respondents who did not engage in any disability prevention activities or who had incomplete data were excluded from the analysis. Participants were classified into two groups: those who implemented disability prevention activities in all three areas and those who implemented at least one but not all three.

**Results:**

Overall, 364 older adults were included in the analysis, of whom 178 (48.9%) implemented disability prevention activities in all three areas. This kind of implementation was significantly associated with the following factors: aged 75-79 years (adjusted odds ratio [aOR] = 2.31), female sex (aOR = 2.13), hypertension (aOR = 0.61), cognitive impairment (aOR = 0.56), and use of mobile applications for disability prevention (aOR = 6.56) (p < 0.05).

**Conclusions:**

These findings identify factors associated with the implementation of multi-area disability prevention activities and underscore the need to reconsider current support strategies.

## Introduction

Disability prevention activities are becoming increasingly crucial as the number of older adults worldwide rapidly increases; therefore, they are particularly promoted in aging societies ^(1), (2), (3)^. For example, these activities, through regular exercise, prevent a decline in physical ^(4), (5)^, cognitive ^(6), (7)^, and activities of daily living ^(8), (9)^ functions and avoid the transition to frailty and the need for care ^(10), (11)^. This positively impacts individual well-being ^(12)^, quality of life ^(13)^, and mortality ^(14)^, and reduces the health care and long-term care costs of the country ^(15), (16)^. However, participation in disability prevention activities is hindered by various barriers, ranging from personal and psychological to environmental factors ^(17), (18), (19), (20)^. Furthermore, it has been suggested that perceptions and impacts of these barriers vary depending on an individual’s activity level ^(19), (20)^. Therefore, it is essential that support for promoting disability prevention activities be based on implementation-related factors, while simultaneously considering differences in individual activity levels.

Exercise is the most prevalent activity for preventing disability; however, combining it with interventions for nutritional status can have a synergistic effect ^(21), (22)^. In contrast, a nutrition-only intervention yields negligible improvement in lower strength performance, underscoring the inadequacy of a single approach for achieving specific goals ^(21)^. Furthermore, improving nutritional status and oral function is beneficial, since oral function is essential in maintaining good nutritional status ^(23), (24)^. Given that these factors influence each other ^(25), (26)^, a deficiency in one area can not only prevent the combined effect but may also weaken the effect on other areas.

The interactions between these domains are complex, with the relationship between oral and physical function often being an indirect effect mediated by nutritional status; reportedly, not only does a decline in the fine motor skills of the tongue and lips increase the risk of frailty via worsened nutritional status ^(23)^, but comprehensive oral hypofunction also leads to inadequate protein intake because specific foods such as meat and beans are avoided ^(24)^. Furthermore, a negative cascade has also been demonstrated in which a lack of occlusal support leads to dysphagia, which in turn precipitates malnutrition and a decline in activities of daily living ^(25)^. In addition, each area has been identified as a factor that affects prognosis ^(27), (28), (29)^, and is essential when considering disability prevention. Thus, preventing disabilities necessitates implementing activities covering multiple aspects of daily function, nutritional status, and oral function. Consistent with these mechanisms, intervention studies using a combined approach that includes physical exercise, nutritional guidance, and oral health instruction have demonstrated improvements in objective indicators of physical and oral function ^(30), (31)^. Furthermore, a comprehensive strategy encompassing all three areas has been reported to be more strongly associated with better activities of daily living outcomes than partial approaches ^(32)^. These findings underscore the clinical advantage of multi-area implementation over interventions focused on fewer areas and highlight the need to develop strategies that support multi-area activities by considering the multifactorial and interconnected nature of frailty.

Nevertheless, the factors associated with implementing multi-area disability prevention activities, as well as the realities of their implementation, remain unclear. In other words, scientific evidence is lacking on how to provide comprehensive support for activities across the key areas of daily function, nutritional status, and oral function. Considering the global trajectory of aging ^(1)^, there is an urgent need to establish strategies to support more meaningful disability prevention activities.

This study aimed to determine the actual conditions and factors associated with the implementation of multi-area disability prevention activities related to daily function, nutritional status, and oral function, as well as to examine factors associated with implementing activities across all three areas. We also sought to identify subgroups that may warrant prioritized outreach and support, thereby providing foundational evidence to inform the design of strategies that promote comprehensive multi-area implementation.

## Materials and Methods

### Participants

In this cross-sectional study, the results of a mail survey conducted among older adults in 10 locations within Hiroshima Prefecture, Japan, were analyzed. The survey was conducted between February and March 2023. The target areas were selected considering the characteristics of each region: urban, rural, and mountainous. The target population was selected using the Basic Resident Ledger Network System, and 1,800 older adults aged ≥65 years were randomly selected from the 10 locations. During the selection process, individuals residing in nursing homes or facilities and those aged <65 years were excluded from the survey. In total, 1,800 older adults were mailed a paper-based self-report questionnaire, and 769 responded.

This study was approved by the Epidemiology Ethics Review Committee of Hiroshima University (Approval No. E2023-0014). The participants were informed of the nature of the study. Written consent for participation was obtained upon return of the questionnaires, and ethical considerations were in accordance with the Declaration of Helsinki.

### Measures

The primary outcome was the total number of areas (daily function, nutritional status, and oral function) in which disability prevention activities were implemented. In this study, “multi-area disability prevention activities” were defined as activities implemented across all three areas. Participants were categorized into two groups: those who implemented disability prevention activities across all three domains of daily function, nutritional status, and oral function (multi-area group) and those who implemented at least one but not all three (two-or-fewer-areas group). The survey content on the actual status of disability prevention activities and related factors was determined through discussions among specialists in medicine, dentistry, oral function, physical therapy, occupational therapy, and nutrition, all of whom hold doctorates.

In this study, disability prevention activities were defined as “activities to reduce the possibility of requiring some form of care and to continue living as independently as possible.” ^(33)^ The questionnaire used for assessment contained questions on the implementation (yes/no) and specific content of disability prevention activities in the domains of daily function, nutritional status, and oral function.

For disability prevention activities related to daily function, participants were initially asked to answer “yes” or “no” to the question, “Are you engaged in disability prevention activities related to daily function?” Those who answered “yes” to this question were subsequently asked to check all items they implemented from the following list: calisthenics, walking, resistance training, sports, stretching, participation in disability prevention classes, and exercise instruction at hospitals and facilities. The implementation rate for each activity was defined as the number of participants who checked a specific activity (e.g., calisthenics) divided by the total number who answered “yes” to engaging in daily functional activities. The rates for the other activities were calculated in the same manner.

Similarly, for disability prevention activities related to nutritional status, participants were initially asked about implementation (yes/no); those who responded “yes” were asked to check all the specific activities they implemented. The implementation rate for each activity was defined using the same calculation method adopted for daily function. The classification of disability prevention activities for nutritional status was as follows: nutritional balancing, low-salt intake, weight control, increased protein intake to match exercise, nutritional guidance at hospitals and facilities, intake of nutritional and food supplements, and active intake of calcium.

Following the same procedure used for the other areas, we assessed oral function-related activities. The implementation rate for each activity was calculated after confirming whether participants engaged in the activity and identifying the specific activities they implemented. The classification of disability prevention activities for oral function was as follows: increasing the number of times they chew, maintaining oral cleanliness, doing oral exercises, doing oral and salivary gland massages, participating in disability prevention classes, and having regular dental checkups.

The following items were selected as variables potentially associated with the total number of areas in which prevention practices were implemented: age, sex, having a spouse and children, living together as a family, presence of caregiving needs, presence of underlying medical conditions, body mass index, access to information, and use of mobile applications for disability prevention. The Kihon Checklist was used to assess various aspects of physical and mental function in older adults. This checklist is a valid and reliable assessment tool for evaluating the functions and abilities associated with frailty in older adults ^(34)^. It has 25 questions and includes the following seven domains: 1-5: Instrumental activities of daily living, 6-10: Physical function, 11-12: Nutritional status, 13-15: Oral function, 16-17: Social isolation, 18-20: Cognitive function, and 21-25: Depression (Supplementary Table 1). Decline or risk in each domain is determined based on the number of negative responses using the following cutoff values: physical function (≥3), nutritional status (≥2), oral function (≥2), social isolation risk (≥1), cognitive function (≥1), depression risk (≥2), and total daily function (≥10).

### Statistical analysis

To describe participants’ characteristics, means and standard deviations were calculated for continuous variables, and numbers and percentages were calculated for categorical variables. Intergroup comparisons were performed using *t*-tests or χ^2^ tests. Furthermore, calculations were made to determine the percentage of each preventive activity implementation content in each group for the actual status of daily function, nutritional status, and oral function. For calculating implementation rates, the denominator for the two-or-fewer-areas group was restricted to participants engaged in at least one activity within the corresponding area. The implementation rates for each activity were compared between the two groups using the chi-square test. Factors associated with implementing disability prevention activities in all three domains were identified using multivariable logistic regression. In the analysis, the dependent variable was defined as whether participants implemented activities in all three areas (1 = multi-area; 0 = two-or-fewer-areas). Univariate logistic regression analyses were performed to estimate crude odds ratios (ORs) and 95% confidence intervals (CIs). Second, a multivariable logistic regression model was constructed to calculate adjusted ORs (aORs). To ensure model interpretability and statistical stability while considering the exploratory nature of this cross-sectional study for identifying associated factors, variables with p-values < 0.25 in the univariate analyses were selected as candidates ^(35), (36), (37)^ and included in the final multivariable model. The goodness-of-fit of the final multivariable model was assessed using the Hosmer-Lemeshow test, and the Akaike Information Criterion was used as a reference. Multicollinearity among independent variables was assessed using the variance inflation factor. All statistical analyses were performed using R version 4.1.3 (R Foundation for Statistical Computing, Vienna, Austria). Significance level was set at 5%.

## Results

### Participants

In this study, 364 participants were included in the analysis. Participants were excluded if they did not engage in disability prevention activities (n = 121), had missing responses regarding age, sex, or the Kihon Checklist (n = 134), or had missing responses regarding disability prevention activities (n = 150). Table 1 presents the participant characteristics of the multi-area and two-or-fewer-areas groups. Overall, 178 participants (48.9%) implemented disability prevention activities in all three areas, whereas 186 participants (51.1%) did not.

**Table 1.** Participant Characteristics.

	Multi-area group	Two-or-fewer-areas group	p-Value
	(n = 178)	(n = 186)	
**Age group, n (%)**			0.103
65-69	32 (18.0%)	40 (21.5%)	
70-74	66 (37.1%)	58 (31.2%)	
75-79	45 (25.3%)	35 (18.8%)	
≥80	35 (19.7%)	53 (28.5%)	
**Sex, male, n (%)**	69 (38.7%)	110 (59.1%)	< 0.001
**Spouse and children, n (%)**			0.304
Has a spouse and children	119 (66.8%)	130 (69.8%)	
Spouse, but no children	10 (5.6%)	17 (9.1%)	
Has children but no spouse	39 (21.9%)	33 (17.7%)	
No spouse and children	10 (5.6%)	6 (3.2%)	
**Family composition living together, n (%)**			0.101
Married Couple living together	85 (47.7%)	91 (48.9%)	
Living alone	37 (20.7%)	24 (12.9%)	
Other	56 (31.4%)	71 (38.1%)	
**Need for care, yes, n (%)**	14 (7.8%)	29 (15.5%)	0.033
**Comorbidities, n (%)**			
Hypertension	80 (44.9%)	111 (59.6%)	0.006
Hyperlipidaemia	32 (17.9%)	28 (15.0%)	0.541
Diabetes	35 (19.6%)	34 (18.2%)	0.839
Osteoarthritis	21 (11.7%)	21 (11.2%)	1.000
Other	47 (26.4%)	41 (22.0%)	0.395
**Body mass index (kg/m^2^), mean ± SD**	23.1 ± 3.3	23.3 ± 3.4	0.539
**Kihon Checklist, n (%)**			
Decline in physical function	42 (23.5%)	51 (27.4%)	0.474
Decline in nutritional status	2 (1.1%)	3 (1.6%)	1.000
Decline in oral function	4 (2.2%)	15 (8.0%)	0.023
Social isolation risk	4 (2.2%)	15 (8.0%)	0.279
Decline in cognitive function	46 (25.8%)	80 (43.0%)	< 0.001
Depression risk	42 (23.5%)	57 (30.6%)	0.163
Decline in total daily function	12 (6.7%)	29 (15.5%)	0.012
**How to obtain information, use, n (%)**			
Newspaper	128 (71.9%)	121 (65.1%)	0.195
TV	158 (88.8%)	150 (80.6%)	0.045
Radio	31 (17.4%)	19 (10.2%)	0.065
Video websites	21 (11.8%)	23 (12.4%)	0.995
Internet	49 (27.5%)	44 (23.7%)	0.467
Magazine	66 (37.1%)	43 (23.1%)	0.005
Advertisement	68 (38.2%)	46 (24.7%)	0.007
Hospital	71 (39.9%)	72 (38.7%)	0.902
Family & Relatives	77 (43.3%)	80 (43.0%)	1.000
Acquaintances & neighbors	81 (45.5%)	63 (33.9%)	0.030
Other	10 (5.6%)	3 (1.6%)	0.075
Total, mean ± SD	4.2 ± 2.3	3.6 ± 1.7	0.001
**Use of mobile applications for disability prevention, yes, n (%)**	38 (21.3%)	7 (3.7%)	< 0.001

Multi-area group: implementation of disability prevention activities across all three areas (daily function, nutritional status, and oral function). Two-or-fewer-areas: implementation of disability prevention activities in two-or-fewer-areas. *p*-Values were calculated using the t-test or chi-square test. SD: standard deviation; TV: television.

### Characteristics of preventive activity patterns in the multi-area and two-or-fewer-areas groups

In the two-or-fewer-areas group (n = 186), the most common area in which any disability prevention activity was implemented was “daily function” (65.0%), followed by “nutritional status” (59.1%), and “oral function” (24.7%) (Supplementary Figure 1). The percentage of those simultaneously implementing activities in multiple areas was 28.4% for “daily function and nutritional status,” 12.3% for “nutritional status and oral function,” and 8.1% for “daily function and oral function.“

**Figure 1.**
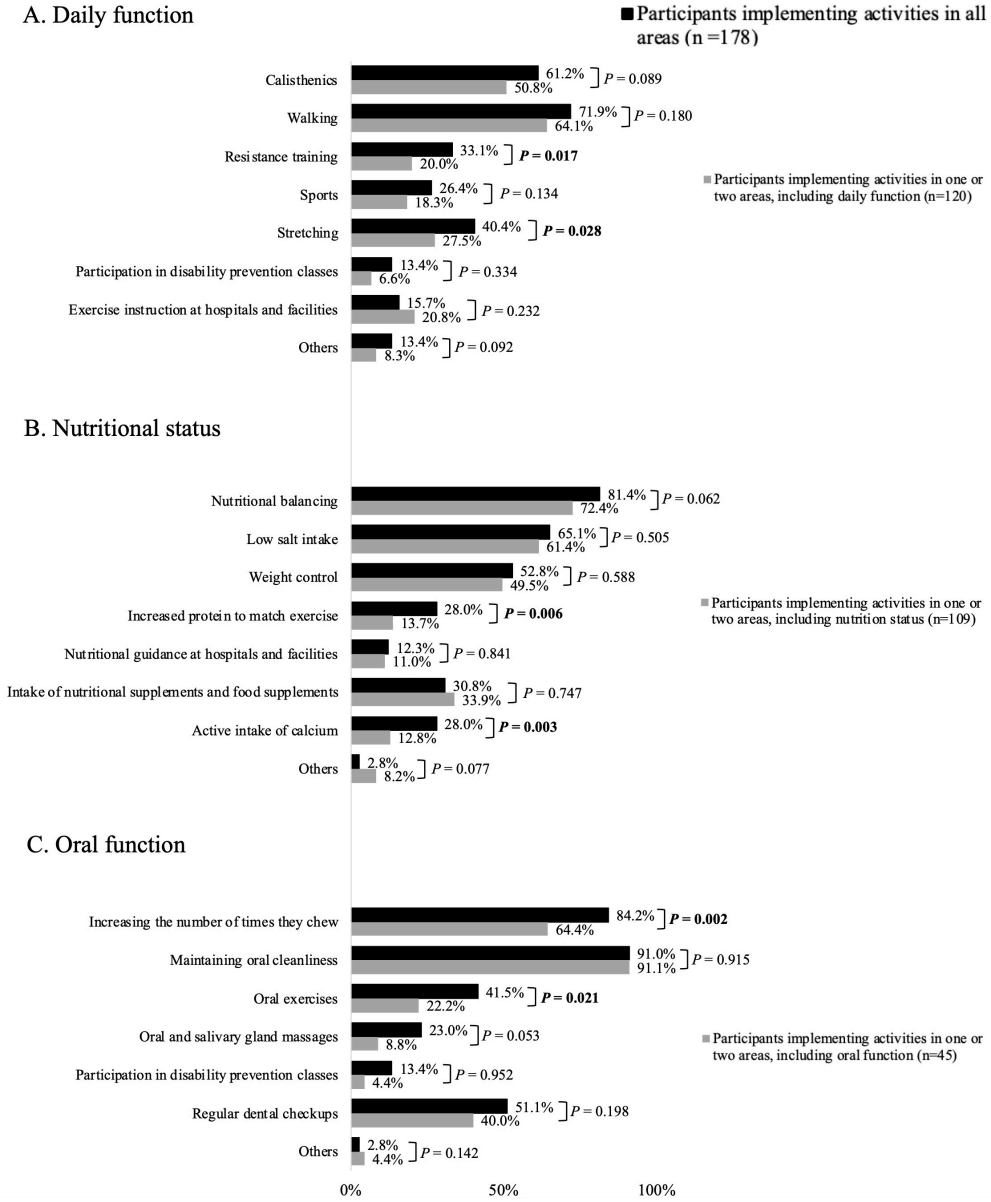
Comparison of implementation rates for specific preventive activities. Group comparisons were performed using the chi-square test. (A) Implementation rates of activities related to daily function. (B) Implementation rates of activities related to nutritional status. (C) Implementation rates of activities related to oral function. Black bars represent the multi-area group (participants implementing activities in all three areas), and gray bars represent the two-or-fewer-areas group (participants implementing activities in two or fewer areas).

Figure 1 shows the implementation rates of specific preventive activities in the multi-area group and the two-or-fewer-areas group. In the daily function area, the implementation rates for “resistance training“ and “stretching“ were significantly higher in the multi-area group (p < 0.05) (Figure 1A). In the nutritional status area, the implementation rates for “increased protein to match exercise” and “active intake of calcium” were significantly higher in the multi-area group than in the two-or-fewer-areas group (p < 0.05) (Figure 1B). In the oral function area, the implementation rates for “increasing the number of times they chew” and “oral exercises” were also significantly higher in the multi-area group (p < 0.05) (Figure 1C).

### Factors associated with the implementation of multi-area disability prevention activities for daily function, nutritional status, and oral function

Table 2 presents the results of the univariate and multivariable logistic regression analyses. The final multivariable model revealed that aged 75-79 years (aOR = 2.31; 95% CI, 1.12-4.82), female sex (aOR = 2.13; 95% CI, 1.32-3.45), and use of mobile applications for disability prevention (aOR = 6.56; 95% CI, 2.84-17.41) were independently and positively associated with the implementation of disability prevention activities in all three areas. Conversely, hypertension (aOR = 0.61; 95% CI, 0.38-0.98) and cognitive impairment (aOR = 0.56; 95% CI, 0.33-0.94) were negatively associated (p < 0.05). No multicollinearity was observed among the independent variables (variance inflation factor range, 1.05-2.17). The Hosmer-Lemeshow test yielded a p-value of 0.787, and the Akaike Information Criterion was 461.73.

**Table 2.** Factors Associated with Multi-Area Disability Prevention Activities.

	Crude odds ratio (95% CI)^a^	p-Value	Adjusted odds ratio (95% CI)^b^	p-Value
**Age group**				
65-69	Ref	Ref	Ref	Ref
70-74	1.42 (0.79-2.56)	0.237	1.58 (0.81-3.13)	0.180
75-79	1.61 (0.85-3.07)	0.147	2.31 (1.12-4.82)	0.024
≥80	0.83 (0.44-1.55)	0.551	1.20 (0.55-2.60)	0.648
**Sex**				
Male	Ref	Ref	Ref	Ref
Female	2.29 (1.51-3.49)	< 0.001	2.13 (1.32-3.45)	0.002
**Spouse and children**				
Has a spouse and children	Ref	Ref		
Spouse, but no children	0.64 (0.27-1.44)	0.290		
Has children but no spouse	1.29 (0.76-2.19)	0.341		
No spouse and children	1.82 (0.66-5.49)	0.260		
**Family composition living together**				
Married Couple living together	Ref	Ref	Ref	Ref
Living alone	1.65 (0.92-3.01)	0.098	1.58 (0.79-3.23)	0.203
Other	0.84 (0.53-1.33)	0.470	0.83 (0.50-1.39)	0.482
**Need for care**	0.46 (0.23-0.89)	0.025	1.07 (0.38-3.07)	0.892
**Hypertension**	0.55 (0.36-0.83)	0.005	0.61 (0.38-0.98)	0.043
**Hyperlipidemia**	1.24 (0.71-2.16)	0.453		
**Diabetes**	1.09 (0.65-1.85)	0.736		
**Osteoarthritis**	1.05 (0.55-2.01)	0.88		
**Body mass index**	0.98 (0.92-1.04)	0.539		
**Kihon Checklist**				
Decline in physical function	0.82 (0.51-1.31)	0.403		
Decline in nutritional status	0.69 (0.09-4.23)	0.690		
Decline in oral function	0.26 (0.07-0.74)	0.019	0.33 (0.08-1.10)	0.088
Social isolation	0.59 (0.25-1.31)	0.204	0.78 (0.28-2.10)	0.618
Decline in cognitive function	0.46 (0.29-0.72)	0.001	0.56 (0.33-0.94)	0.031
Depression	0.70 (0.44-1.11)	0.132	0.93 (0.50-1.71)	0.805
Decline in total daily function	0.39 (0.19-0.78)	0.009	0.92 (0.29-2.81)	0.882
**Total number of sources of information**	1.19 (1.07-1.33)	0.002	1.13 (1.00-1.28)	0.055
**Use of mobile applications for disability prevention**	6.94 (3.19-17.4)	< 0.001	6.56 (2.84-17.41)	< 0.001

The dependent variable was implementation of disability prevention activities in all three areas (1 = multi-area; 0 = two-or-fewer-areas). ^a^Univariate logistic regression analysis. ^b^Multivariable logistic regression analysis (Hosmer-Lemeshow test, p = 0.787; Akaike Information Criterion = 461.73). CI: confidence interval; Ref: reference.

## Discussion

The results of this study showed that aged 7579 years, female sex, absence of hypertension, absence of cognitive impairment, and use of mobile applications for disability prevention were associated with implementing multi-area disability prevention activities for daily function, nutritional status, and oral function. The conditions and factors associated with implementing activities in a single area have been previously investigated ^(17), (18), (19), (20)^. However, to our knowledge, the actual conditions of multi-area activities, which effectively prevent disabilities, and the factors associated with their implementation, remain to be clarified.

Our results revealed that approximately half of the older adults who were engaged in disability prevention do not perform multi-area activities for daily function, nutritional status, and oral function. It can be inferred that older adults who are already engaged in one or more activities are more likely to be motivated and interested in disability prevention ^(38)^ and are less likely to be resistant to adding areas of disability prevention. In other words, the current results indicate that there are older adults for whom the effectiveness of disability prevention activities could be further enhanced.

Focusing on the relevant factors identified in this study may lead to more efficient and effective support for daily function, nutritional status, and oral function. Since digital devices such as applications are valuable modifiable factors amenable to intervention ^(39), (40)^, leveraging these tools to provide personalized information is a particularly promising strategy for promoting multi-domain activities that lead to disability prevention. Furthermore, female sex was also associated with multi-area implementation, which may reflect sex differences in health consciousness and behavior ^(41), (42)^. The finding that a significant association with age was observed exclusively in the 75-79-year age group suggests that the relationship between aging and activity implementation is not linear. This age range may represent a suitable life stage for engaging in multi-area activities, as social constraints such as employment decrease ^(43)^, whereas functional reserve remains relatively preserved compared with that in adults aged 80 years and older, who are more prone to frailty ^(44)^. In contrast, the high prevalence of frailty among individuals aged 80 years and older ^(44)^ makes disability prevention particularly critical. However, the specific reasons for the lack of an association in this age group remain unclear and should be addressed in future studies. Conversely, a history of hypertension and cognitive decline was identified as a barrier to multi-area implementation. Cognitive decline may pose challenges to multi-area disability prevention, as it can directly impair the management, planning, and execution of behaviors ^(45), (46)^. In addition, hypertension not only imposes limitations on physical exertion ^(47)^, but its higher prevalence among men ^(48)^ and individuals with cognitive decline ^(49)^ may also involve complex interrelationships among these factors.

Furthermore, many of the factors identified in this study as associated with the implementation of these prevention activities are components of health literacy ^(50)^ or are related to it ^(51)^. This finding suggests that health literacy may be a common underlying factor for each of these associated factors. Previous studies have shown that health literacy is associated with health promotion activities ^(52)^, access to health information ^(53)^, and physical activity ^(54)^. Health literacy may be associated with the number of disability prevention domains implemented. Notably, comprehensive factors, including subtypes of health literacy, were not measured in this study; meanwhile, this point may serve as a meaningful hypothesis for further advancing how disability prevention activities are supported.

Characteristic patterns were identified in the implementation of disability prevention activities between the multi-area and two-or-fewer-areas groups. In the two-or-fewer-areas group, the implementation rate of activities related to oral function was markedly lower than that for daily function and nutritional status; interestingly, approximately 30% of this group simultaneously engaged in activities for daily function and nutrition. This discrepancy suggests that, compared with general exercise and dietary improvements, knowledge ^(55)^ and recognition ^(56)^ regarding the importance of maintaining and improving oral function remain low. However, disability and frailty progress through multifactorial mechanisms. Therefore, the three domains (daily function, nutritional status, and oral function) should be viewed as interconnected rather than independent entities. Specifically, oral hypofunction impairs mastication and swallowing, reducing food diversity and intake, leading to nutritional deterioration ^(57), (58)^. Poor nutritional status subsequently contributes to functional decline and incident disability ^(59), (60)^. Furthermore, reduced daily function limits social participation, which has been reported to be related to oral function ^(61)^. Consequently, effective disability prevention requires a comprehensive approach that encompasses all three domains to interrupt this cycle and strengthen preventive strategies ^(62), (63)^. Consistent with this comprehensive approach, the multi-area group had significantly higher implementation rates of evidence-based activities ^(64), (65), (66)^, such as resistance training and protein intake combined with exercise. This finding suggests that the multi-area group was more likely to engage in effective preventive strategies, whereas individuals implementing activities in two or fewer areas may require support to integrate existing habits with the often neglected oral health components.

This study has practical and clinical implications for the prevention of disability in community-dwelling older adults. Identifying factors associated with multi-area activity implementation provides valuable insights for planning and delivering targeted outreach and support, as well as for designing pathways that facilitate access to information and promote participation. Specifically, the observed associations can serve as a reference for practitioners to identify subgroups that are less likely to engage in comprehensive multi-area activities, thereby aiding in the prioritization of additional and more personalized approaches. Furthermore, this study suggests that expanding implementation to encompass all three areas represents a challenge distinct from initiating participation in disability prevention activities. Accordingly, designing support systems that bridge these gaps may offer a promising approach to fostering comprehensive engagement. Although causal inference is limited by the cross-sectional study design, these findings may contribute to the future development of strategies aimed at preventing frailty and reducing the need for long-term care among community-dwelling older adults by providing a foundation for hypothesis generation.

This study has some limitations. First, the study was limited to a single prefecture in Japan, possibly limiting its generalizability to other prefectures with regional characteristics and countries outside Japan. However, Hiroshima Prefecture is often described as a microcosm of Japan, as it possesses geographical diversity and has a population composition close to the national average ^(67)^. Furthermore, participants were randomly selected from 10 locations (urban, rural, and mountainous areas) to minimize bias in regional characteristics. These points may mitigate the limitation regarding generalizability. However, data on specific residential characteristics were not collected. Previous studies have shown that the built environment, including walkability, is associated with physical activity and social participation among older adults ^(68)^ and that terrain- and region-specific environmental factors can influence the risk of physical inactivity ^(69)^. Accordingly, unmeasured residential environmental factors may have influenced activity implementation in this study. Future research should incorporate residential data and objective environmental indicators to better clarify contextual factors that may facilitate or hinder the implementation of activities across these three domains. Second, the survey was cross-sectional; therefore, it is limited in its ability to estimate causal relationships. Furthermore, variables for the multivariable model were selected based on preceding analyses. Though this approach is useful for identifying potentially relevant factors, its data-driven model composition means the results should be cautiously interpreted as highlighting important predictors. Thus, future longitudinal and intervention studies are required to confirm the findings of this study. Third, incomplete responses were excluded to ensure the reliability of the survey results. Selection bias may exist, as those whose responses were complete may have had a relatively high interest in disability prevention activities. Therefore, caution should be taken when interpreting the results.

In conclusion, our results showed the actual conditions and factors associated with implementing multi-area disability prevention activities for these three domains among community-dwelling older adults. These findings identify factors associated with multi-area disability prevention activities and provide new insights into the nature of support methods for their implementation.

## Article Information

### Acknowledgments

This study was conducted as part of the Home Rehabilitation Support Program of the Family of Older Adults Requiring Long-term Care, sponsored by the Hiroshima Prefectural Association of Medical and Care Facilities. We express our deepest gratitude to the following committee members for their contributions to this study: Takeshi Kikutani (Nippon Dental University); Ryoichi Yasui (Kojika Iryou Ryouiku Center); Shusaku Kanai (Prefectural University of Hiroshima); Jun Kayashita (Prefectural University of Hiroshima); Kota Munetsugu (Hiroshima Cosmopolitan University); Hirofumi Yamaji (Hiroshima International University); and Yuki Ishihara (BeRISE, Inc.). We would also like to express our deepest gratitude to the Survey Research Center, Inc. Hiroshima Office and all participants who provided their cooperation in the survey.

### Author Contributions

All authors contributed to the planning of the study design, development of the questionnaire, interpretation of results, writing of the article, and approval of the final manuscript. SI corresponded with the research company for data acquisition. KN, HJ, HO, and SI were involved in data analysis and preparation of the manuscript.

### Conflicts of Interest

None

### IRB Approval Code and Name of the Institution

This study was approved by the Epidemiology Ethics Review Committee of Hiroshima University (Approval No. E2023-0014). The participants were informed of the nature of the study, written consent for participation was obtained upon return of the questionnaires, and ethical considerations were made according to the Declaration of Helsinki.

### Data Availability Statement

The datasets analyzed in this study are not publicly available for privacy and personal information.

## Supplement

Supplementary Material
