# Supplementary material for: Actual Conditions and Implementation-Related Factors of Activities to Prevent Disability in Multi-Area of Daily Function, Nutritional Status, and Oral Function among Community-Dwelling Older Adults
Source: JMA J. 2026 Mar 27;9(3):617–27. doi: 10.31662/jmaj.2025-0558 (PMC13246259; doi:10.31662/jmaj.2025-0558)
Supplement: Supplementary Material [file 2433-3298-9-3-0617-s001.pdf]

Supplementary Table 1. The Kihon Checklist

No.	Questions	Answer	
1	Do you go out by bus or train by yourself?	0. YES	1. NO
2	Do you go shopping to buy daily necessities by yourself?	0. YES	1. NO
3	Do you manage your own deposits and savings at the bank?	0. YES	1. NO
4	Do you sometimes visit your friends?	0. YES	1. NO
5	Do you turn to your family or friends for advice?	0. YES	1. NO
6	Do you normally climb stairs without using handrail or wall for support?	0. YES	1. NO
7	Do you normally stand up from a chair without any aids?	0. YES	1. NO
8	Do you normally walk continuously for 15 minutes?	0. YES	1. NO
9	Have you experienced a fall in the past year?	1. YES	0. NO
10	Do you have a fear of falling while walking?	1. YES	0. NO
11	Have you lost 2 kg or more in the past 6 months?	1. YES	0. NO
12	Height: cm, weight: kg, BMI: kg/m2 If BMI is less than 18.5, this item is scored.	1. YES	0. NO
13	Do you have any difficulties eating tough foods compared to 6 months ago?	1. YES	0. NO
14	Have you choked on your tea or soup recently?	1. YES	0. NO
15	Do you often experience having a dry mouth?	1. YES	0. NO
16	Do you go out at least once a week?	0. YES	1. NO
17	Do you go out less frequently compared to last year?	1. YES	0. NO
18	Do your family or your friends point out your memory loss?	1. YES	0. NO
19	Do you make a call by looking up phone numbers?	0. YES	1. NO
20	Do you find yourself not knowing today's date?	1. YES	0. NO

21	In the last 2 weeks have you felt a lack of fulfilment in your daily life?	1. YES	0. NO
22	In the last 2 weeks have you felt a lack of joy when doing the things you used to enjoy?	1. YES	0. NO
23	In the last 2 weeks have you felt difficulty in doing what you could do easily before?	1. YES	0. NO
24	In the last 2 weeks have you felt helpless?	1. YES	0. NO
25	In the last 2 weeks have you felt tired without a reason?	1. YES	0. NO

Note: Working Group on Frailty in Japanese Geriatrics Society. BMI, body mass index.

Reference:

Arai H, Satake S. English translation of the Kihon Checklist. Geriatr Gerontol Int. 2015 Apr;15(4):518-9. doi: 10.1111/ggi.12397. Erratum in: Geriatr Gerontol Int. 2022 Feb;22(2):187. doi: 10.1111/ggi.14351.

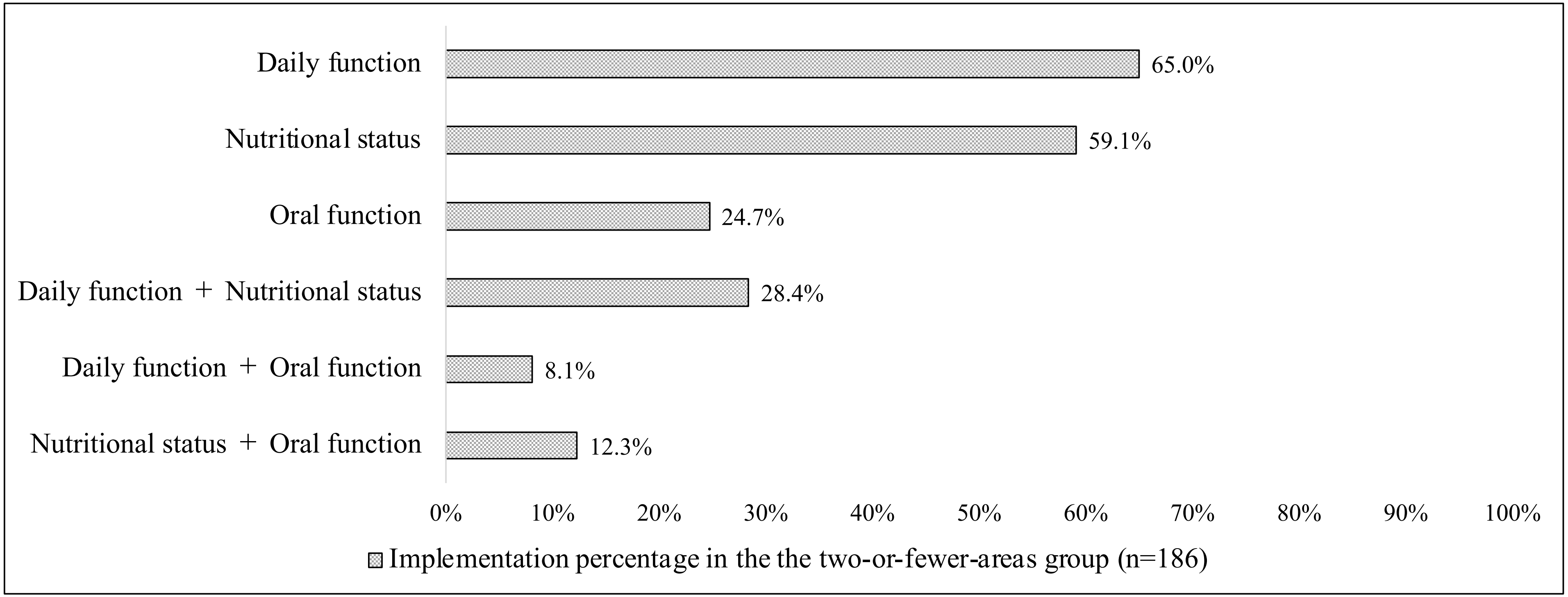

Supplementary Figure 1. Implementation percentage of each disability prevention area in the two-or-fewer-areas group
